# Impact of race‐specific screening guideline on the uptake of colorectal cancer screening among young African Americans

**DOI:** 10.1002/cam4.4842

**Published:** 2022-05-29

**Authors:** Hyo Jung Tak, I‐Wen Pan, Michael T. Halpern, Ya‐Chen Tina Shih

**Affiliations:** ^1^ Department of Health Services Research and Administration University of Nebraska Medical Center Omaha NE USA; ^2^ Department of Health Services Research University of Texas MD Anderson Cancer Center Houston TX USA; ^3^ Healthcare Delivery Research Program National Cancer Institute Bethesda MD USA

**Keywords:** cancer screening, colorectal cancer, racial disparities, U.S. multi‐society task force

## Abstract

**Background:**

African Americans (AAs) have had lower colorectal cancer (CRC) screening rates, higher incidence rate, and earlier mean age at onset. The 2017 U.S. Multi‐Society Task Force (MSTF) recommended initiating CRC screening at age 45 for AAs and age 50 for non‐AAs.

**Objective:**

To investigate the impact of the 2017 MSTF's race‐specific guidelines on CRC screening rate among young AAs.

**Design, setting, and participants:**

We used the 2015 and 2018 National Health Interview Survey to provide nationally representative estimates. The study sample included adults aged between 45 and 75 without a history of CRC, excluding screening recipients for diagnosis or surveillance purposes.

**Main measures:**

The outcome is a binary variable of CRC screening. Primary independent variables were age and race category (non‐AAs aged 45–49, AAs 45–49, non‐AAs 50–75, AAs 50–75), a binary variable indicating before or after the 2017 MSTF guideline (2015 vs. 2018), and their interaction terms. We employed a multivariable logistic model, adjusting for individual characteristics, and accounting for complex survey design.

**Key results:**

Among the total sample (*n* = 21,735), CRC screening rate increased from 54.6% in 2015 to 58.5% in 2018 (*p* < 0.01). By age and race, the screening rate exhibited an increase for all age and race groups except for young non‐AAs. Compared to young non‐AAs, the adjusted predicted probability (APP) of screening for young AAs was significantly higher by 0.10 (average marginal effect, 0.10; 95% confidence interval, 0.01–0.19) in 2018, while the difference was insignificant in 2015. Racial differences in screening among older adults were not significant in both years. The CRC screening rate was substantially lower among young AAs compared to older AAs (17.2% vs. 65.5% in 2018).

**Conclusion:**

The race‐specific recommendation is an effective policy tool to increase screening uptake and would contribute to reducing cancer disparities among racial/ethnic minorities.

## Graphical abstract

This study used the 2015 and 2018 National Health Interview Survey to investigate the impact of the 2017 U.S. Multi‐Society Task Force's race‐specific guidelines on colorectal cancer screening rate among young African Americans. Compared to young non‐African Americans, the adjusted predicted probability of colorectal cancer screening for young African Americans was significantly higher in 2018, implying that the race‐specific recommendation is an effective policy tool to increase screening uptake and reduce cancer disparities among racial/ethnic minorities.

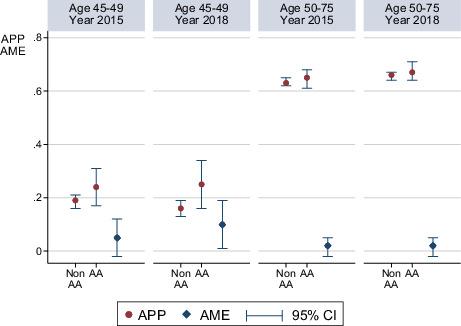

## 1 INTRODUCTION

Colorectal cancer (CRC) is the third most common malignancy and the third leading cause of cancer deaths among U.S. adults.^1^ The effectiveness of CRC screening in the detection of precancerous lesions and reduction in mortality is well established in the literature,^2^, ^3^ making CRC screening participation a priority in cancer prevention and control effort. Despite various national campaigns to promote CRC screening,^4^, ^5^ approximately one‐quarter of U.S. adults at screening‐eligible age have not been screened as recommended in 2018^6^ and the racial disparities in CRC screening persisted.^7^, ^8^

CRC incidence and mortality rates have decreased in the past a few decades due to increased and improved screening, reduced exposure to CRC risk factors, advanced treatments, and policy interventions.^1^, ^9^, ^10^, ^11^ However, this clinical improvement has been observed primarily among individuals aged 50 years or older.^12^, ^13^ CRC has depicted a puzzling trend in young adults, with disproportionately increasing incidence in individuals younger than 50 years since the mid‐1990s^13^, ^14^, ^15^, ^16^ and slightly increasing mortality in individuals younger than 55 years since the mid‐2000s.^17^ Furthermore, African Americans (AAs) have had lower CRC screening rates, higher incidence rates, earlier mean age at onset, and higher mortality.^12^, ^18^, ^19^, ^20^, ^21^

Awareness of increasing trend of CRC incidence among young adults^13^, ^14^, ^15^, ^16^ and the higher disease burden of CRC in AAs^12^, ^18^, ^19^, ^20^, ^21^ call for a closer examination to determine whether a racial‐specific recommendation for optimal age of CRC screening initiation is warranted among the average‐risk population. In response, the U.S. Multi‐Society Task Force of Colorectal Cancer (MSTF) released one of the few race‐specific screening guidelines in 2017, in which they lowered the initiation age of CRC screening to 45 for AAs and kept the screening initiation age at 50 for non‐AAs with average risk of CRC.^18^ While the American College of Gastroenterology (ACG) issued a similar recommendation in 2009,^22^ the impact on boosting screening uptake among AAs aged between 45 and 49 would likely be limited as it was from a single professional society.^23^ In contrast, the 2017 MSTF recommendations are expected to be more authoritative than the ACG guidelines because this represents consensus from three gastroenterology societies—the ACG, the American Gastroenterological Association, and the American Society of Gastrointestinal Endoscopy, and opportunely corresponds to growing concerns over the increasing incidence of CRC among younger adults.

This study examines whether the 2017 MSTF CRC screening guidelines were associated with an increase in CRC screening uptake among young (age 45–49 years) AAs. Understanding the impact of the 2017 MSTF guidelines on screening behaviors between race groups is a critical first step to assess the potential of mitigating cancer disparities through screening recommendations targeted at vulnerable subgroups.

## 2 METHODS

### 2.1 Data sources and study population

We used the 2015 and 2018 National Health Interview Survey (NHIS).^24^ The NHIS data are cross‐sectional household surveys of non‐institutionalized civilians in the United States. The sampling frame is guided by rigorous survey design to produce nationally representative estimates.

The annual survey consists of several modules. For this study, we retrieved information on history of cancer diagnosis and screening for various cancers from the 2015 NHIS Sample Adult Cancer module and 2018 NHIS Sample Adult module. We also used Family, Person, and Family Income modules to obtain individual‐level information, such as demographics, socioeconomic status, risk factors of CRC incidence, general health status, and health services utilization, and family‐level information such as family income.

**FIGURE 1:**
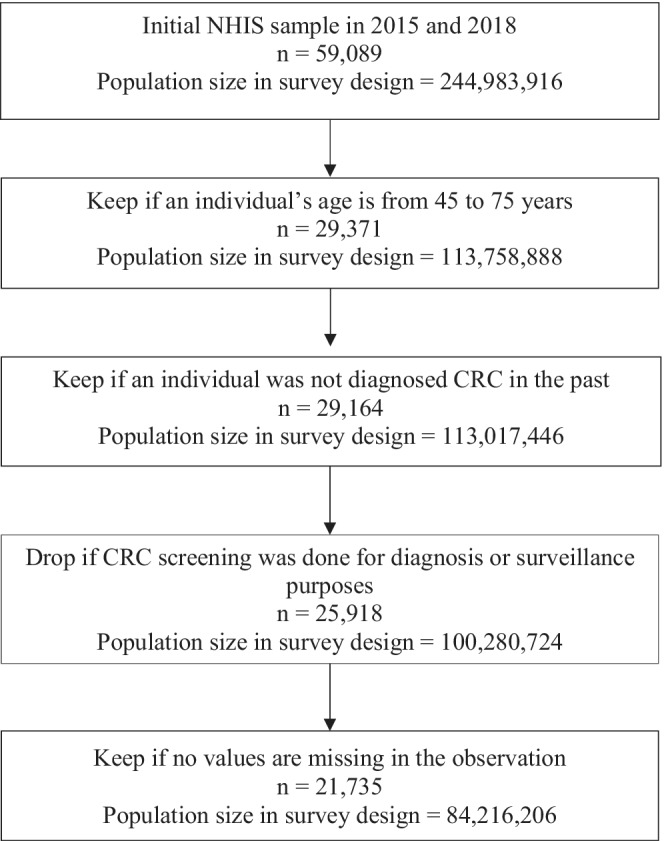
Applicable Study Population Selection Criteria for Colorectal Cancer (CRC) Screening in the 2015 and 2018 National Health Interview Survey (NHIS)

Figure 1 depicts the inclusion/exclusion criteria used to construct our study population. Briefly, we included patients who (i) were between 45 and 75 years old, and (ii) had no CRC diagnosis in the past. We excluded individuals who received CRC screening for diagnosis or surveillance purposes rather than part of routine exam. We restricted the upper age of our study population to 75 because most CRC screening guidelines, including the 2017 MSTF guidelines, recommend screening up to age 75.

This study was exempt from the requirements of the Institutional Review Board at the University of Nebraska Medical Center.

### 2.2 Data elements

#### 2.2.1 Outcome

The outcome is a binary variable indicating whether an individual had ever received CRC screening via any of the following screening modalities: colonoscopy, sigmoidoscopy, CT colonoscopy, blood stool or FIT test, and Cologuard®.

#### 2.2.2 Primary independent variables

Primary independent variables were a categorical variable combining age and race groups (young non‐AAs, young AAs, older non‐AAs, older AAs), a binary variable (YEAR) indicating before and after the 2017 MSTF guidelines (2015, YEAR = 0 vs. 2018, YEAR = 1), and interaction terms between age‐race groups and year. Young adults included the age cohort newly added in the 2017 MSTF guidelines (age 45–49 years), while older adults included the age from 50 to 75. AAs included non‐Hispanic and Hispanic blacks, and non‐AAs included all other racial and ethnic groups.

#### 2.2.3 Other independent variables

Other variables in the multivariable regression analyses included gender, employment status, family income category, and primary insurance status (private, Medicare, Medicaid and other, and no insurance). As individuals with higher CRC risk factors would be more likely to be recommended for CRC screening,^25^, ^26^ we added smoking status (never smoked, former smoker, current sometime smoker, and current everyday smoker), obesity measured by body mass index (BMI) (underweight or normal [BMI < 25], overweight [25 ≤ BMI < 30], and obese or extreme obese [BMI ≥30]), and frequency of light to moderate physical activity (never/unable, 0–2 times, 3–5 times, and ≥ 6 times per week on average). We also adjusted for general health status (fair or poor, good, and very good or excellent), chronic diseases (three binary variables for diabetes, hypertension, and chronic obstructive pulmonary disease each), usual source of care, and region (Northeast, Midwest, South, and West). Having a usual source of care was quantified as a binary variable, indicating an individual had a usual place to visit when one was sick and the place was not a hospital outpatient department or a hospital emergency room.

### 2.3 Statistical analysis

For bivariate analyses, we used Pearson Chi‐squared tests to examine whether there was systematic difference in each covariate by CRC screening status, and to compare the CRC screening rate by age and age‐race groups. We also restricted the sample to young adults aged between 45 and 49 and compared the covariates between non‐AAs and AAs. Then we employed multivariable logistic regression model to investigate the effect of the 2017 MSTF guidelines on CRC screening rate, adjusting for all other explanatory variables described above.

Due to the non‐linearity of logistic regressions, an interaction term in logistic regression models cannot be interpreted in the same way that interaction terms are explained in linear regressions.^27^ To improve interpretability of the impact of a race‐specific screening guideline on the uptake of CRC screening by race, we first derived adjusted predicted probability (APP) of CRC screening by age and race category and year from the multivariable logistic model. We then derived average marginal effects (AME) for each age cohort (age 45–49 years vs. age 50–75 years) and year (2015 vs. 2018) to assess the difference in the APP of CRC screening between non‐AAs and AAs.

We performed two sensitivity analyses. First, we removed the upper age of the study population. Second, we quantified physical activity using the survey question on the frequency for vigorous activity instead of light to moderate activity.

All statistical analyses accounted for complex survey design (i.e., individual probability weight, primary sampling unit, and strata) to generate nationally representative estimates. All statistical tests were two‐sided and *p*‐values ≤0.05 were considered statistically significant. All analyses were performed by Stata MP v.16.1 (StataCorp LLC, College Station, TX).

## 3 RESULTS

### 3.1 Descriptive statistics

The total sample size was 21,735, which included 12,183 adults in 2015 and 9552 adults in 2018, respectively. This represents 84,216,206 U.S. adults (41,801,972 in 2015 and 42,414,234 in 2018) who met the study population inclusion/exclusion criteria. Table 1 describes the characteristics of the study population and compares them by CRC screening status. Among the total study population, 18.2% were young adults (45–49 years), 11.7% were AAs, 48.4% were women, 63.6% were employed, and 53.1% and 22.9% were covered by private insurance and Medicare, respectively. For health risk factors, 11.9% smoked every day, 36.0% were obese or extremely obese, and 38.4% did not do any light to moderate physical activity per week on average. Furthermore, 15.3% rated their health status as fair or poor and 49.0% had at least one chronic disease. Comparisons between non‐AAs and AAs among young adults (age 45–49) are shown in Table 2.

**TABLE 1:** Demographics, Socioeconomic Status, Health Risk Factors of Colorectal Cancer, Health Status, Usual Source of Care, and Region among Total Study Population and Stratified by Colorectal Cancer Screening Status (*n* [%])

	Total study	Colorectal cancer screening		
	population	No	Yes	
	(*n* = 21,735)	(*n* = 9221)	(*n* = 12,514)	*p*‐value
Age category				<0.001
45–49 (young adults)	3416 (18.2)	3020 (36.6)	396 (4.1)	–
50–75 (older adults)	18,319 (81.8)	6201 (63.4)	12,118 (95.9)	–
Race				<0.001
Non‐African Americans	18,823 (88.3)	7924 (87.6)	10,899 (88.8)	–
African Americans	2912 (11.7)	1297 (12.4)	1615 (11.2)	–
Year				<0.001
2015	12,183 (49.5)	5512 (51.8)	6671 (47.8)	–
2018	9552 (50.5)	3709 (48.2)	5843 (52.2)	–
Women	10,019 (48.4)	4338 (48.5)	5681 (48.3)	0.708
Employed	13,004 (63.6)	6330 (72.3)	6674 (57.0)	<0.001
Family income category				<0.001
Bottom quartile	5447 (17.7)	2784 (21.0)	2663 (15.1)	–
Second quartile	5586 (23.4)	2395 (24.6)	3191 (22.4)	–
Third quartile	5648 (28.1)	2137 (25.8)	3511 (29.8)	–
Top quartile	5054 (30.9)	1905 (28.7)	3149 (32.7)	–
Insurance status				<0.01
Private insurance	10,188 (53.1)	4898 (59.3)	5290 (48.3)	–
Medicare	5845 (22.9)	1183 (9.9)	4662 (33.0)	–
Medicaid and other	4033 (16.3)	1785 (16.6)	2248 (16.1)	–
No insurance	1669 (7.7)	1355 (14.3)	314 (2.7)	–
Smoking status				<0.001
Never smoker	12,086 (58.1)	5163 (59.6)	6923 (56.9)	–
Former smoker	6049 (27.0)	1956 (20.4)	4093 (32.1)	–
Current sometime smoker	716 (3.0)	414 (3.9)	302 (2.4)	–
Current everyday smoker	2884 (11.9)	1688 (16.0)	1196 (8.7)	–
Obesity				0.191
Underweight or normal	6308 (28.1)	2770 (29.0)	3538 (27.5)	–
Overweight	7639 (35.9)	3193 (35.3)	4446 (36.3)	–
Obese or extreme obese	7788 (36.0)	3258 (35.7)	4530 (36.2)	–
Physical activity				<0.001
Never or unable	8547 (38.4)	4123 (43.0)	4424 (34.9)	
0–2 times per week on average	3851 (18.5)	1550 (17.7)	2301 (19.1)	
3–5 times per week on average	4410 (20.6)	1619 (18.6)	2791 (22.1)	
≥6 times per week on average	4927 (22.5)	1929 (20.7)	2998 (23.9)	
General health status				0.730
Fair or poor	3650 (15.3)	1591 (15.1)	2059 (15.4)	–
Good	6435 (29.8)	2760 (30.1)	3675 (29.5)	–
Very good or excellent	11,650 (54.9)	4870 (54.7)	6780 (55.1)	–
Diabetes	3485 (15.4)	1164 (11.8)	2321 (18.1)	<0.001
Hypertension	9883 (43.6)	3339 (34.6)	6544 (50.4)	<0.001
COPD	1137 (4.6)	337 (3.1)	800 (5.8)	<0.001
Usual source of care	19,055 (88.3)	7301 (80.4)	11,754 (94.3)	<0.001
Region				<0.001
Northeast	3761 (18.6)	1382 (16.4)	2379 (20.3)	–
Midwest	4686 (21.5)	1909 (20.7)	2777 (22.0)	–
South	7835 (37.9)	3467 (39.6)	4368 (36.7)	–
West	5453 (22.0)	2463 (23.3)	2990 (21.1)	–

*Notes*: (i) COPD: chronic obstructive pulmonary disease. (ii) Percentages were adjusted for probability weight, primary sampling unit, and strata in survey design analysis.

**TABLE 2:** Demographics, Socioeconomic Status, Health Risk Factors of Colorectal Cancer, Health Status, Usual Source of Care, and Region among Young Adults (Age 45–49) and Stratified by Race (*n* [%])

	Young	Race		
	adults only	Non‐AAs	AAs	
	(n = 3416)	(n = 2936)	(n = 480)	p‐value
Race				–
Non‐African Americans (AAs)	2936 (87.4)	2936 (100.0)	0 (0.0)	–
African Americans (AAs)	480 (12.6)	0 (0.0)	480 (100.0)	–
Year				0.524
2015	2032 (51.1)	1723 (50.8)	309 (53.2)	–
2018	1384 (48.9)	1213 (49.2)	171 (46.8)	–
Women	1601 (49.8)	1395 (49.9)	206 (49.2)	0.811
Employed	2847 (84.8)	2478 (85.4)	369 (80.8)	0.042
Family income category				<0.001
Bottom quartile	674 (13.6)	529 (12.3)	145 (22.6)	–
Second quartile	777 (20.7)	621 (19.5)	156 (29.1)	–
Third quartile	901 (27.0)	792 (26.8)	109 (28.4)	–
Top quartile	1064 (38.7)	994 (41.4)	70 (19.9)	–
Insurance status				<0.001
Private insurance	2395 (73.3)	2118 (74.7)	277 (63.6)	–
Medicare	0 (0.0)	0 (0.0)	0 (0.0)	–
Medicaid and other	556 (13.6)	430 (12.3)	126 (22.7)	–
No insurance	465 (13.1)	388 (13.1)	77 (13.8)	–
Smoking status				0.133
Never smoker	2184 (66.4)	1838 (65.8)	346 (70.8)	–
Former smoker	626 (18.5)	567 (19.1)	59 (14.0)	–
Current sometime smoker	126 (3.4)	111 (3.2)	15 (4.2)	–
Current everyday smoker	480 (11.7)	420 (11.8)	60 (11.0)	–
Obesity				<0.001
Underweight or normal	949 (26.9)	858 (28.2)	91 (17.9)	–
Overweight	1196 (36.3)	1026 (36.0)	170 (38.5)	–
Obese or extreme obese	1271 (36.8)	1052 (35.8)	219 (43.6)	–
Physical activity				0.555
Never or unable	1278 (36.8)	1067 (36.6)	211 (38.2)	–
0–2 times per week on average	713 (21.9)	623 (22.2)	90 (19.6)	–
3–5 times per week on average	712 (20.7)	627 (20.9)	85 (19.2)	–
≥6 times per week on average	713 (20.6)	619 (20.2)	94 (23.0)	–
General health status				<0.001
Fair or poor	442 (11.2)	342 (10.5)	100 (16.4)	–
Good	949 (28.9)	804 (28.1)	145 (34.4)	–
Very good or excellent	2025 (59.8)	1790 (61.4)	235 (49.1)	–
Diabetes	279 (7.8)	241 (8.0)	38 (6.7)	0.396
Hypertension	919 (25.7)	728 (23.8)	191 (38.9)	<0.001
COPD	66 (1.7)	59 (1.8)	7 (1.1)	0.321
Usual source of care	2805 (83.0)	2421 (83.3)	384 (80.5)	0.222
Region				<0.001
Northeast	536 (16.4)	461 (16.3)	75 (17.2)	–
Midwest	719 (20.8)	632 (21.3)	87 (17.8)	–
South	1230 (38.2)	951 (35.4)	279 (57.7)	–
West	931 (24.5)	892 (27.0)	39 (7.3)	–

*Notes*: (i) COPD: chronic obstructive pulmonary disease. (ii) Percentages were adjusted for probability weight, primary sampling unit, and strata in survey design analysis.

**FIGURE 2:**
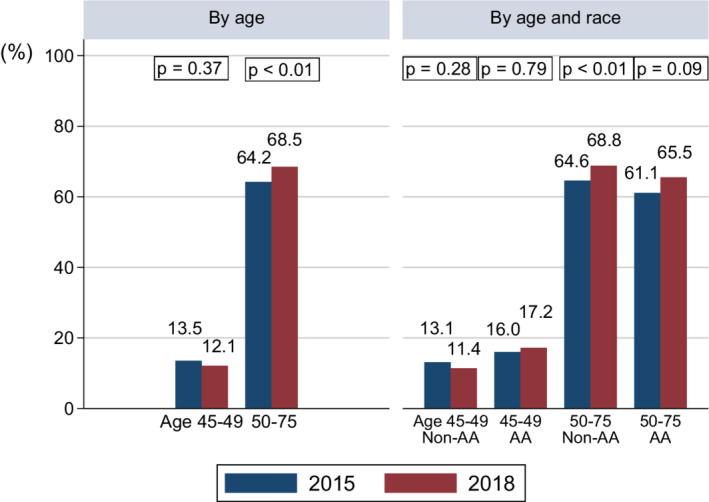
Colorectal Cancer Screening Rate by Age, and Age and Race (*n* = 21,735) *Notes*: (i) AA: African Americans. (ii) Percentages were adjusted for probability weight, primary sampling unit, and strata in survey design analysis

The CRC screening rate increased from 54.6% in 2015 to 58.5% in 2018 (*p* < 0.01). Among those who have ever received CRC screening, the proportion of young adults was disproportionately lower (4.1%). When stratified by age group, the CRC screening rate increased significantly among older adults (*p* < 0.01) but was not significantly different among young adults between 2015 and 2018. By age and race, CRC screening rates were higher in 2018 among all age‐race cohorts except for young non‐AAs, but statically significant increase was observed only among the older non‐AAs (Figure 2).

### 3.2 Multivariable logistic regression model

**FIGURE 3:**
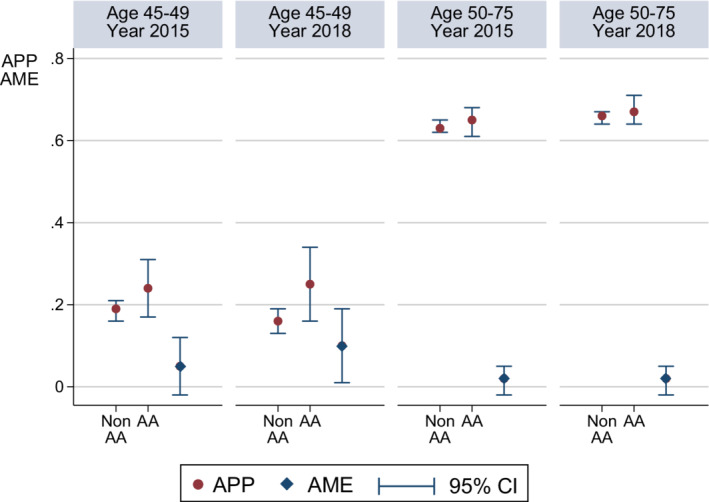
Impact of the 2017 U.S. Multi‐Society Task Force of Colorectal Cancer (MSTF) Guideline Updates on Colorectal Cancer Screening: By Age and Race Before and After the MSTF Recommendation (*n* = 21,735) *Notes*: (i) AA: African Americans. (ii) Adjusted predicted probability (APP) of CRC screening and average marginal effect (AME: difference in APP between non‐AA and AA) were adjusted for all other explanatory variables listed in Table 1 in multivariable logistic regression. (iii) APP, AME, and 95% confidence interval (CI) were adjusted for probability weight, primary sampling unit, and strata in survey design analysis to generate nationally representative estimates. (iv) APP and AME are statistically significant at *p* ≤ 0.05 if its 95% CI does not include zero

Figure 3 illustrates the APP of CRC screening and AME derived from the multivariable logistic regression. For young adults (age 45–49), the APP of CRC screening in 2015 was 0.19 (95% confidence interval [CI], 0.16–0.21) and 0.24 (95% CI, 0.17–0.30) for non‐AAs and AAs, respectively. The difference in APP between these two race groups in 2015 was not statistically significant (AME, 0.05; 95% CI, −0.02 to 0.12). For young adults, the APP of CRC screening in 2018 was 0.16 (95% CI, 0.13–0.19) and 0.25 (95% CI, 0.16–0.34) for non‐AAs and AAs, respectively. The difference in the APP of CRC screening by race in 2018 was statistically significant, with the APP 0.10 higher for AAs (AME, 0.10; 95% CI, 0.01–0.19). For older adults (age 50–75), the differences in APP of CRC screening between non‐AAs and AAs were not statistically significant in either 2015 or 2018.

Estimates in multivariable logistic regression showed that individuals who were employed (odds ratio [OR], 0.77; 95% CI, 0.69–0.85) and uninsured (OR, 0.43; 95% CI, 0.35–0.52, compared to private insurance) were less likely to receive CRC screening whereas those with higher family income and a usual source of care were more likely to undergo CRC screening. Compared to individuals who never smoked, former smokers were more likely to receive CRC screening (OR, 1.33; 95% CI, 1.20–1.47) while individuals who currently smoke every day (OR, 0.60; 95% CI, 0.52–0.68) were less like to receive it. Although obesity is a risk factor of CRC, it was not statistically significantly associated with CRC screening. Physical activity and chronic conditions were positively associated with CRC screening. In addition, individuals residing in the South and West were significantly less likely to undergo CRC screening than those in Northeast (Table 3).

**TABLE 3:** Impact of the 2017 U.S. Multi‐Society Task Force of Colorectal Cancer Guideline Updates on Colorectal Cancer Screening (*n* = 21,735)

	OR (95% CI)
Age and race category	
45–49 (young adults), non‐African Americans	1.00
45–49 (young adults), African Americans	1.40 (0.88, 2.20)
50–75 (older adults), non‐African Americans	9.67 (7.86, 11.9)
50–75 (older adults), African Americans	10.5 (8.21, 13.4)
Year category	
2015	1.00
2018	0.82 (0.62, 1.10)
Interaction between age and race and year 2018	
45–49 (young adults), non‐African Americans	1.00
45–49 (young adults), African Americans	1.34 (0.65, 2.73)
50–75 (older adults), non‐African Americans	1.41 (1.04, 1.90)
50–75 (older adults), African Americans	1.46 (1.01, 2.11)
Women	1.09 (1.00, 1.19)
Employed	0.77 (0.69, 0.85)
Family income category	
Bottom quartile	1.00
Second quartile	1.35 (1.19, 1.55)
Third quartile	1.85 (1.61, 2.11)
Top quartile	2.22 (1.91, 2.58)
Insurance status	
Private insurance	1.00
Medicare	2.32 (2.07, 2.60)
Medicaid and other	1.28 (1.13, 1.46)
No insurance	0.43 (0.35, 0.52)
Smoking status	
Never smoker	1.00
Former smoker	1.33 (1.20, 1.47)
Current sometime smoker	0.72 (0.56, 0.92)
Current everyday smoker	0.60 (0.52, 0.68)
Obesity	
Underweight or normal	1.00
Overweight	1.02 (0.92, 1.14)
Obese or extreme obese	0.97 (0.86, 1.08)
Physical activity	
Never or unable	1.00
0–2 times per week on average	1.52 (1.34, 1.73)
3–5 times per week on average	1.46 (1.30, 1.64)
≥6 times per week on average	1.39 (1.25, 1.56)
General health status	
Fair or poor	1.00
Good	0.99 (0.86, 1.13)
Very good or excellent	1.03 (0.89, 1.19)
Diabetes	1.19 (1.05, 1.36)
Hypertension	1.31 (1.19, 1.44)
Chronic obstructive pulmonary disease	1.78 (1.43, 2.20)
Usual source of care	2.60 (2.25, 2.99)
Region	
Northeast	1.00
Midwest	0.93 (0.81, 1.08)
South	0.85 (0.74, 0.97)
West	0.82 (0.71, 0.95)

*Notes*: odds ratio (OR) and 95% confidence interval (CI) were adjusted for probability weight, primary sampling unit, and strata in survey design analysis.

Findings from each sensitivity analysis were similar to those from the main analyses, both in terms of the magnitude and statistical significance of estimates.

## 4 DISCUSSION

The MSTF updated its CRC screening guidelines in 2017, specifically recommending lowering the screening initiation age to 45 for AAs. This study used the 2015 and 2018 NHIS data to assess the impact of the 2017 MSTF guidelines, focusing on the young adults aged between 45 and 49 who were recommended to receive the CRC for the first time in their lives per the 2017 guidelines. We found the adjusted predicted CRC screening rate among young adults (age 45–49) was significantly higher for AAs compared to non‐AAs in 2018 but not in 2015. This suggests that the race‐specific recommendation in the 2017 MSTF guidelines was effective in increasing the uptake of CRC screening for young AAs.

Racial disparities in CRC screening have been persistent despite various efforts and interventions in public health.^28^, ^29^ CRC screening guidelines underwent several updates in the past two decades,^14^, ^18^, ^22^, ^28^, ^30^, ^31^, ^32^, ^33^, ^34^ with the most recent update published by the U.S. Preventive Services Task Force (USPSTF) in May 2021.^30^ Although the vast majority of CRC screening guidelines acknowledged higher incidence and mortality among AAs, only a few guidelines contained race‐specific recommendations, such as the guidelines from the ACG in 2009,^22^ the Institute for Clinical Systems Improvement (ICSI) in 2010,^34^ and the MSTF in 2017.^18^

Millien and colleagues used 2005, 2010, and 2015 NHIS data to evaluate the impact of CRC screening guidelines released between 2005 and 2015 that contained race‐specific recommendation.^23^ The authors compared CRC screening rate between Whites and AAs aged 45–49, and concluded that guidelines recommending earlier age of screening initiation for AAs were not effective given that there was no difference in screening rate by race in each year. We re‐assessed the impact of race‐specific recommendation based on the 2017 MSTF guidelines. The MSTF represents their collective efforts and consensus from three gastroenterology societies at a time with immense interests in curbing CRC incidence in young adults and alleviating racial disparities in cancer.^18^ Notably, clinicians have become increasingly aware of the rising incidence of CRC among young adults after the publication of a number of epidemiology studies in the 2010s.^13^, ^14^, ^15^, ^16^ The 2017 MSTF guidelines offered a narrow window of opportunity to assess the latest impact of race‐specific recommendation because two subsequent CRC screening guidelines released by the American Cancer Society in 2018 and the USPSTF in 2021 lowered the initiation of CRC screening to age 45 for all average‐risk but did not differentiate it by race.

Extensive literature has documented racial disparities in CRC.^28^, ^29^, ^35^, ^36^, ^37^, ^38^, ^39^ For example, CRC incidence and mortality rate of AAs was 19.1% and 36.0% higher than non‐Hispanic Whites, respectively.^1^ Researchers have also identified potential reasons for racial disparities in CRC screening stemmed from patients, medical providers, and health care systems. Patient barriers among AAs included poor knowledge of CRC risk, fear of pain during colonoscopy, lower perceived benefit of CRC screening, lower compliance with physicians' CRC screening recommendations, and cancer fatalism.^28^, ^29^ Provider‐level factors, such as poor understanding of patients' barriers, insufficient counseling and outdated knowledge of guideline recommendations, can hinder policy efforts to increase screening uptake. Despite various public interventions and campaigns to mitigate racial disparities in CRC screening,^4^, ^5^, ^28^, ^29^ primary care physicians (PCPs)’ awareness of race‐specific guidelines has been limited. For example, a national physician survey conducted in 2009 showed that only 28.0% of family practitioners, internists, and gastroenterologists correctly identified 45 as the age to screen AAs after the release of the 2008 ACG guidelines.^40^ Although a physician survey conducted in 2019 showed an increasing percentage of PCPs were aware of the guideline updates that lowered the CRC screening initiation age to 45 and also endorsed such practice,^41^ the rate of awareness remained low (38.1%). Finally, researchers have expressed opinions that systematic racial disparities are attributable to individual socioeconomic circumstances, social norms, and health systems rather than biological characteristics itself.^42^, ^43^ Thus, the clinical and policy attentions should be redirected accordingly to mitigate racial disparities, and may explain the absence of race‐specific recommendations from the USPSTF. However, this position is not universally agreed upon among all professional societies and the academic community, as evident from a recent commentary advocating for specific breast cancer screening recommendations for Black women.^44^

Race‐specific recommendations in screening guidelines can be a powerful tool to endorse the importance of CRC screening and thereby to mitigate racial disparities in cancer screening. Improving physicians' understanding on patients' barriers as well as their awareness on changes in updated guidelines, especially those with race‐specific recommendations, may further increase the CRC screening rate among young AAs. However, empirical evidence related to race‐specific screening guidelines was very limited and most of these studies found that previous race‐specific guidelines were not effective.^23^, ^33^, ^45^ Our evaluation of the 2017 MSTF guidelines contributes to the limited literature on this topic, showing that the race‐specific recommendation was effective in increasing the CRC screening rate among average‐risk young AAs. Our finding could reflect a more favorable policy environment in the last decade as the U.S. health care system has experienced seismic changes during this period. For example, policies such as Affordable Care Act (ACA) and Medicaid expansion have improved minority patients' access to and affordability of cancer care.^46^, ^47^

The MSTF guidelines tackle the racial disparity in CRC screening by lowering the screening initiation age for AAs. This has increased the CRC screening rate among young AAs in 2018, but their screening rate remains much lower than that of older AAs (17.2% vs. 65.5%). Notably, close to 10% of invasive CRC was diagnosed in individuals younger than 50 years.^1^ Therefore, policy efforts to improve early initiation of screening are critical for CRC to prevent more years of life lost from cancer, especially for AAs.

The lower screening rate among young adults may be derived from financial concerns. The ACA prevention provision that waives cost‐sharing requirement applies to USPSTF's Grade A or B recommendations only,^48^ and the USPSTF Grade B recommendation of CRC screening for individuals aged 45–49 was not issued until 2021.^49^ Therefore, young AAs seeking CRC screening following the MSTF recommendation prior to this might have incurred copayments of $750 or higher for colonoscopy.^50^ Indeed, among those who received colonoscopy in our study sample from the 2018 NHIS, 46.6% and 31.6% of young and older adults paid part or all of the cost out of pocket, respectively, suggesting a higher financial burden of CRC screening for young adults. Furthermore, among young adults, non‐AAs were more likely to pay part or all of the cost out of pocket than AAs in 2018 (47.6% vs. 39.6%). Future research should explore the extent to which providers were able to reference the 2017 MSTF guidelines to wave the co‐sharing requirement for young AAs who underwent colonoscopy for CRC screening.

Some limitations of this study deserve discussion. First, several important determinants of CRC screening were not consistently available in the NHIS data. For example, biological family's CRC history is likely a strong predictor of CRC screening^28^ but this information was collected in the 2015 NHIS only. Second, we investigated the effect of the race‐specific recommendation in the 2017 MSTF guidelines by assessing the association of CRC screening by age and race before and after the MSTF guidelines. Although we have accounted for various individual characteristics in the multivariable analysis, there may be time‐variant confounders unaccounted, such as increasing public awareness of CRC risk among young adults. Third, we did not employ the difference‐in‐differences (DiD) model, which is commonly used empirical method to assess the difference in outcomes by age and race groups before and after a policy recommendation or an intervention. This is because the trends of CRC screening rate observed across age and race cohorts in the pre‐ and post‐period violated the parallel trend assumption required for the DiD method.^51^ Lastly, we may have misclassified colonoscopy or blood stool tests performed for diagnosis or surveillance purposes as screening despite the inclusion and exclusion criteria instituted.

In summary, this study used the 2017 MSTF CRC screening guidelines to highlight the potential impact of race‐specific recommendation to increase screening among racial minorities. To optimize the benefit of screening, efforts in public health should go beyond screening and be extended to timely follow‐ups, treatments, and surveillance. The findings from our study would be the first crucial step in the sequence of these efforts to improve CRC screening rates and reduce incidence and mortality rates among young adults, especially young AAs.

## COMPETING INTERESTS

The authors declare that they have no competing interests.

## AUTHOR CONTRIBUTIONS

Dr. Tak had full access to all the data in the study and takes responsibility for the integrity of the data and the accuracy of the data analysis. All authors made substantial contributions to the manuscript and attest to the validity and legitimacy of the data, as well as its interpretation. There was no additional contributor who was not listed as an author.

## CONFLICT OF INTEREST

The funder had no role in design, conduct, and writing of this manuscript or the decision to submit it for publication. Dr. Shih served as a consultant for a review panel for Pfizer Inc. and an advisory board for AstraZeneca in 2019. All other authors have no conflicts of interest to disclose on the subject of this manuscript.

## DISCLAIMER

The views expressed here are those of the authors and do not represent any official position of the National Cancer Institute or the National Institutes of Health.

## ETHICS APPROVAL

This study was exempt from the requirements of the Institutional Review Board at the University of Nebraska Medical Center.

## Data Availability

The data that support the findings of this study are openly available in National Center for Health Statistics at https://www.cdc.gov/nchs/nhis/index.htm, reference number 24.
